# On the Recent Advances in the Theory of Vision

**Published:** 1872-03

**Authors:** H. Helmholtz

**Affiliations:** The Royal Society of London


ART. V.—*On the Recent Advances in the Theory of Vision.* *By* H. HELMHOLTZ, *(of the Royal Society of London.)*

Translated from *Les Annales d'Oculistique*, BY F. W. ABBOTT, M. D.

(*Continued.*)

In the first part of our exposition we followed the progress of the luminous rays as far as the retina, and we saw how the peculiar arrangement of the optical apparatus causes the light which proceeds from every separate luminous point to unite upon a separate point of this membrane, so that each point of the external world irritates only one single terminal nervous apparatus. Ancient physiology did not think it necessary to look further. The external light touching the sensitive nervous substance, they considered that it was immediately perceived.

During the last century, and especially during the first quarter of this, the knowledge of the phenomena of the nervous system made such progress that, in 1826, Jean Muller, then of Bonn, and afterwards of Berlin, was able to write his work on the Comparative Physiology of the Sense of Sight, a work which made an epoch in science, and in which he established principles upon which rests essentially the study of the sensations. Not only have these principles been confirmed as to essential points by later researches, but their import has been found to be greater than the celebrated Berlin philosopher could have supposed from the facts which were known at that time. The propositions of Muller are everywhere cited under the title of *laws of the specific energies of the senses.* These propositions are therefore neither so new nor so little known that it is necessary to speak of them in connection with the recent advances of the theory of vision, and so much the more as they have often been referred to, as well by others as by myself. Nevertheless, as the whole of that part of the theory of vision which now occupies our attention is little else than a development and a consequence of the theory of the specific energies of the senses, I will ask permission to repeat here several things already known, so as to be able to establish a connection between that which I have to say that is new, and that which the reader already knows.

All our perceptions of the external world rest upon the fact, that certain modifications, produced in our organs of sense by the impressions which external objects cause in them, are conducted to the brain by the nerves; it is then only that we are conscious of them, and that they combine so as to furnish representations of objects. If we cut the conducting nerve so as to intercept the road by which the impression reaches the brain, sensation and perception instantly cease. With reference to the eye, the proof that perception does not reside immediately in the two retinas, but that it is formed in the brain, and by means of the impressions which the retinas send to this organ, is furnished by the fact that the representation of relief is only obtained—we will demonstrate this later—by the aid of the combination and the fusion of the images received by both eyes.

Thus, what we immediately perceive is never the influence of the external agent upon the extremities of our nerves, but always a modification transmitted by them, and it is to this modification that we give the name of excitation or irritation.

Now, according to all the facts yet known, all the nervous fibres appear to have the same structure, and the modification to which we give the name of irritation behaves in the very same manner in all, in spite of the great variety of functions fulfilled by the nerves. In truth, it is not their sole function to convey to the brain the sensations of the external organs; others conduct the volitions of the brain to the muscles whose contraction then moves the limbs; others make the action of the brain felt upon certain glands whose secretions they excite, or upon the heart and vessels to regulate the circulation of the blood, etc. The fibres of these nerves, however, are all similar cylindrical threads of a microscopic fineness, transparent as glass, all containing the same contents analogous partly to oil and partly to the white of an egg. If these threads show differences in their diameters, this appears to be connected with circumstances of only secondary importance, such as the question of solidity, or the necessity of the existence of a sufficient number of independent conduits, without the thickness being essentially in proportion to the variety of the functions. All these fibres have the same electro-motor properties as certain researches, especially those made by du Bois Raymond, have demonstrated; in all the condition of irritation may be obtained by the same mechanical, electrical, chemical, or calorific changes, and it is propagated in each sense with the same rapidity, (about one hundred and ten feet a second) and then produces the same changes in their electro-motor properties. Finally, all the fibres die in consequence of the same conditions, and the coagulation of their contents appears to vary only a little by reason of their thickness. In a word, barring the action of the other organs of the body with which they are connected, and where the effect of their irritation is manifested during life, all that is true for one nerve is absolutely applicable to all the others. Still farther, very recently two French physiologists, Messrs. Philippeau and Vulpian, after having cut the sensitive and motor nerves of the tongue, have succeeded in making the upper part of the sensitive nerve grow to the lower end of the motor nerve. The irritation of the upper part, which in normal conditions produces a sensation, was then carried to the motor nerve and to the muscular fibres which it animates, and manifested itself under the form of a motor irritation.

Hence we draw this conclusion, that the diversity of the effects produced by the irritation of the different trunks depends solely on the diversity of the corresponding organs to which the nerve reports its state of irritation.

The nerve fibres have often been compared to the wires of a telegraphic system, and, in truth, this comparison is extremely well chosen to set forth a remarkable and important peculiarity of these fibres. In the different parts of a telegraphic circuit the same copper and iron wires conduct the same kind of motion, the electric current, nevertheless we see the most varied effects produced at the different stations, according to the apparatus with which the wires connect. Sometimes it is a clock that strikes, sometimes it is a dial-plate, a printing, or an electro-chemical telegraph which is put in operation. The shocks which the electric current gives to our members may equally serve as telegraphic signals, and at the time of the laying of the Atlantic cable, Mr. W. Thompson found that the feeblest signals could be recognized by the sensation of taste which was produced by placing the wires upon the tongue. In other cases we employ telegraphic wires to explode mines by the use of strong electric currents. In a word, a telegraphic wire terminating in any point whatever may occasion every one of the effects so innumerably varied which electric currents are capable of producing, and, in spite of the diversity of the effects, it is always the same thing which passes along the wire.

That which precedes is well calculated to show that *when the conditions change, the same causes may produce different effects.* However commonplace this proposition may appear, it is only very slowly, and after great efforts, that we have come to formulate it, and reject the adage according to which the same causes are reputed to produce the same effects. We can scarcely pretend that the application of this proposition is even yet perfectly familiar to us; particularly in the subject with which we are concerned, has the resistance to admitting the consequences of this proposition endured to very recent times.

While the irritation of nerves which terminate in muscles or glands produces motions or secretions, that of the sensitive nerves produces sensations. But we have very different kinds of sensations. First of all, the sensations relating to objects of the external world divide themselves into five completely independent groups. These groups correspond to the five senses, whose difference is so great that it is impossible to make a qualitative comparison between a sensation of light and another sensation, either auditory or olfactory. We call this difference "difference of *modality*," and it is much more profound than that which separates comparable qualities; the expression "qualitative differences" will serve, on the contrary, to designate the differences between sensations pertaining to the same sense, such as, for example, the difference between the sensations of different colors.

If the irritation of a nervous trunk produces a muscular motion, a secretion, or a sensation, this depends solely upon the muscular, glandular, or sensitive nature of the organ where the nerve which is under consideration ends. The effect depends not at all upon the nature of the irritant employed, whether it is an electric shock, torsion, section, irritation by contact with a solution of sea-salt, or cauterization with a red hot iron. We know likewise,—and we owe this immense progress in our knowledge to Jean Muller,—that when we irritate a sensitive nerve the luminous, tactile, olfactory, or gustatory modality does not depend at all upon the kind of irritation, but solely upon the sense to which the irritated nerve appertains.

Let us apply this theory to the optic nerve. In the first place we know that no kind of action upon any part of the body, except the eye and its optic nerve, can ever produce a sensation of light. We take the liberty of doubting the histories of somnambulists, which are alone in contradicting this assertion. On the other hand, external light has not the exclusive privilege of producing a luminous sensation in the eye. Everything which can irritate a nerve may produce this sensation. The feeblest electric currents conducted through the eye produces flashes of light. A blow, or even a slight pressure exerted by the nail upon the lateral part of the eye produces, in the most complete darkness, luminous sensations which, in favorable conditions, may attain very great intensity. It is necessary to remark, that in this experiment no objective light is developed in the retina, as certain physiologists suppose. For the different luminous sensations may be sufficiently intense, for the illumination of the retina, necessary for the production of this sensation, to be easily perceptible to a second observer looking into the eye of the first, if the sensation is truly the consequence of a production of light in the retina. Now no such thing is seen. A pressure or an electric current irritates the optic nerve and, conformably to the law of Muller, there results a luminous sensation; but, at least under the conditions of experiment, they are incapable of producing the least trace of objective light.

So also, a congestion, or an alteration in the composition of the blood, which may be produced by certain febrile states, or the injestion of intoxicating or narcotic substances may produce, in the visual nervous apparatus, luminous sensations to which no external light corresponds. Finally, in cases of complete absence of the eye, in consequence of a wound or an operation, the compression exerted by the cicatrix upon the nervous trunk may still produce fantastic luminous sensations.

From what precedes it follows principally, that the peculiar modality by which the luminous sensation is distinguished from all others does not reside in qualities peculiar to the external light, to which this sensation can respond, but that, far from this, every thing which can irritate the optic nerve induces a sensation so like that produced by external light, that those ignorant of the law of this phenomenon easily believe in the presence of a veritable objective light.

External light, therefore, produces upon the optic nerve nothing besides that which may be produced by agents of any other nature. In one single respect is light favored in comparison with the other means of irritation: the optic nerve, situated behind the eyeball at the bottom of the bony cavity of the orbit, is almost entirely shut away from the influence of every other means of irritation, which only exceptionally touch it, while the rays of light may always reach it without obstacle, by traversing the transparent media of the eye. On the other hand, owing to the rods and cones, these terminal organs of its fibres, the optic nerve is infinitely more sensitive to the rays of light than are any other of the nervous apparatuses of the body, which perceives rays of light only when these rays are sufficiently concentrated to produce a sensible increase of temperature.

This circumstance explains how the irritation of the ocular nervous apparatus has become for us the ordinary sensual sign which discloses the presence of light in the field of vision, and how we do not distinguish between light and the luminous sensation even when the latter alone exists. But in considering the facts as a whole, we discover, beyond a doubt, that ths external light is only *one* of the irritations which may act upon the optic nerve, and that there exists no exclusive relation between light and the luminous sensation.

After having spoken of the general influence of irritations upon the nerves of sensation, we pass to the study of the qualitative differences of the luminous sensations, that is to say, to the differences in the sensations produced by the different colors, and let us apply ourselves particularly to finding out in what measure the differences of these sensations correspondent to real or objective differences.

*To be Continued.*

